# Mobile Helpline and Reversible Contraception: Lessons From a Controlled Before-and-After Study in Rural India

**DOI:** 10.2196/12672

**Published:** 2019-08-09

**Authors:** Sangita Kulathinal, Bijoy Joseph, Minna Säävälä

**Affiliations:** 1 Department of Mathematics and Statistics University of Helsinki Helsinki Finland; 2 Akra-Numero Ltd Helsinki Finland; 3 The Family Federation of Finland (Väestöliitto) Helsinki Finland; 4 Social and Cultural Anthropology Faculty of Social Sciences University of Helsinki Helsinki Finland

**Keywords:** contraception behavior, family planning services, organizations, nonprofit, cell phone use, mobile phone, information seeking behavior, mHealth, call center, South Asia, India

## Abstract

**Background:**

Researchers and activists have expressed concerns over the lack of availability and nonuse of reversible, modern, contraceptive methods in India for decades. New attempts to increase access, availability, and acceptance of reversible contraceptives need to be developed, instead of relying solely on female sterilization. Mobile health (mHealth) initiatives may offer one way to serve underprivileged populations who face challenges in sexual and reproductive health (SRH) in countries such as India.

**Objective:**

This study aimed to examine the outcome of an mHealth intervention for enhancing knowledge of, and practices related to, reversible contraceptives in rural Western India.

**Methods:**

We implemented a nonrandomized controlled trial (before-and-after study in an intervention area and a control area) in the Indian state of Maharashtra. The intervention in this case was a mobile-based SRH helpline provided by a nongovernmental organization (NGO). Baseline and follow-up surveys were carried out in two government-run primary health center areas, one each in the intervention and control area, and 405 respondents were surveyed in the two rounds. An interview-based structured questionnaire suitable for a low-literacy environment was used to collect data. The effect of the intervention was estimated using logistic regression, adjusted for gender, by calculating robust standard errors to take into account the clustering of individuals by the area (intervention or control). In each regression model, the effect of intervention was estimated by including a term for interaction between the intervention area and the period before and after the intervention. The exponent of the regression coefficient of the interaction term corresponding to the period after the intervention, along with the 95% CI, is reported here. The odds ratio for the control village multiplied by this exponent gives the odds ratio for the intervention village. Calls received in the intervention were recorded and their topics analyzed.

**Results:**

The current use of reversible contraception (18% increase in intervention area vs 2% increase in control area; 95% CI) has seen changes. The proportion of respondents who had heard of contraception methods from an NGO rose in the intervention area by 23% whereas it decreased in the control area by 1% (95% CI). However, the general level of awareness of reversible contraception, shown by the first contraceptive method that came to respondents’ mind, did not improve. Demand for wider SRH information beyond contraception was high. Men and adolescents, in addition to married women, made use of the helpline.

**Conclusions:**

A mobile helpline that one can confidentially approach at a time most convenient to the client can help provide necessary information and support to those who need reversible contraception or other sexual health information. Services that integrate mHealth in a context-sensitive way to other face-to-face health care services add value to SRH services in rural India.

## Introduction

### Background

In India, a few reproductive mobile health (mHealth) initiatives have been implemented [1-6], of which the most popular is the Central Government’s Mother and Child Tracking System [7]. However, the use of mHealth in family planning and contraceptive services has thus far been limited. In addition, research evidence on mHealth interventions and programs in the field of sexual and reproductive health (SRH) in India remains scarce, although mHealth offers great promise to potentially help cater to the needs of people with limited health care and family planning services [4,5,7]. Telemedicine, emergency services, text messaging services, supervision and support services to the health care service staff, and data collection are among the functions that mobile phones have brought in to improve reproductive health services in less developed countries [5,8].

With its population of 1.3 billion people, India has implemented the Indian family planning program, which relies heavily on female sterilization. Of currently married women, aged 15 to 49 years, 36% are sterilized whereas 11% use reversible modern contraception [9]. Reversible modern contraceptive methods in India mainly include condoms, intrauterine contraceptive devices (IUCDs), oral contraceptive pills and injectable contraceptives. Researchers and activists have expressed concern over the lack of availability and nonuse of reversible modern contraceptive methods in India since decades. The Indian Government expressed its changing focus toward reversible methods in 2012 [10] by expanding the choice of methods, especially encouraging the postpartum adoption of IUCDs. It is expected that increased use of reversible contraceptive methods will help in reducing both maternal and infant mortality and morbidity, as well as slow down population growth by lengthening birth intervals.

The use of reversible contraception remains abysmally low in India. Among the underlying structural factors are the generally low socioeconomic and educational standards [11], and gender and generational asymmetries [12-15]. Practically, lacking information on reversible contraceptive methods or fears related to side effects [16], inaccessibility or poor quality of care [17], and provider-imposed barriers [18] hinder adoption of contraception, and even more so adoption of reversible methods.

Consequently, there is a need to provide personal counseling and information in a gender-sensitive manner, secure access to contraceptives and improve health care services, particularly among the socioeconomically underprivileged groups. Can mHealth assist in providing counseling and information and improve accessibility of contraception? Wireless phone subscribers in India reached a total of 998 million people by March 2018 [19], and mobile phones are increasingly affordable and accessible even among the poorest in rural India [20]. Mobile technology is thus a potential means to also reach out to the disadvantaged.

### Purpose of the Study

This study explores gaps in SRH needs of rural, disadvantaged populations, and further examines the outcome of an mHealth intervention in enhancing knowledge and practice of reversible contraception to reduce the gap in rural Western India. An mHealth-based intervention on family planning is examined in a nonrandomized controlled trial (before-and-after study in an intervention area and a control area) in the Indian state of Maharashtra. The intervention was a project by a nongovernmental organization (NGO) offering mobile-based SRH helpline.

## Methods

### Study Setting

In terms of family planning, the Indian state of Maharashtra represents close to the average Indian situation. In the state, 51% of currently married women are sterilized whereas 11.5% use reversible modern contraceptive methods of which the condom is the most popular. Total unmet need for contraception is 9.7% [21].

Overall, two sufficiently similarly profiled districts, Thane and Nashik of Maharashtra, were chosen for the study. Although Thane is more urban than Nashik, the subdistricts where primary health center (PHC) areas were chosen for the data collection have a similar level of urbanization (77% in the chosen subdistrict within Thane and 78% in the chosen subdistrict within Nashik [22]). Overall sociodemographic characteristics and contraceptive use patterns were similar enough in the two districts. A PHC area in rural Thane was the intervention area and a PHC area in rural Nashik was the comparable nonintervention area which provided the control group for the study.

### Intervention

The intervention introduced a mobile helpline, combined with personal contact with participants by village health workers and with local distribution of contraceptives. The target population was married men and women in the age range of 15 to 35 years.

A toll-free helpline was available to villagers in the project area. A total of 12 gender-equal, frontline, field workers were responsible for communication and branding activities for the call center, and follow-up activities among targeted beneficiaries who sought the intervention services, using both interpersonal communication and mid-media activities (eg, street theater and wall paintings). The calls were attended by trained female and male paramedical staff, one of each, who were fluent in the local dialect. The helpline personnel recorded the received calls, specifically noting the main topic of discussion. This intervention complemented the regular governmental health care services available, and it took place from June 2015 to June 2016.

**Figure 1:**
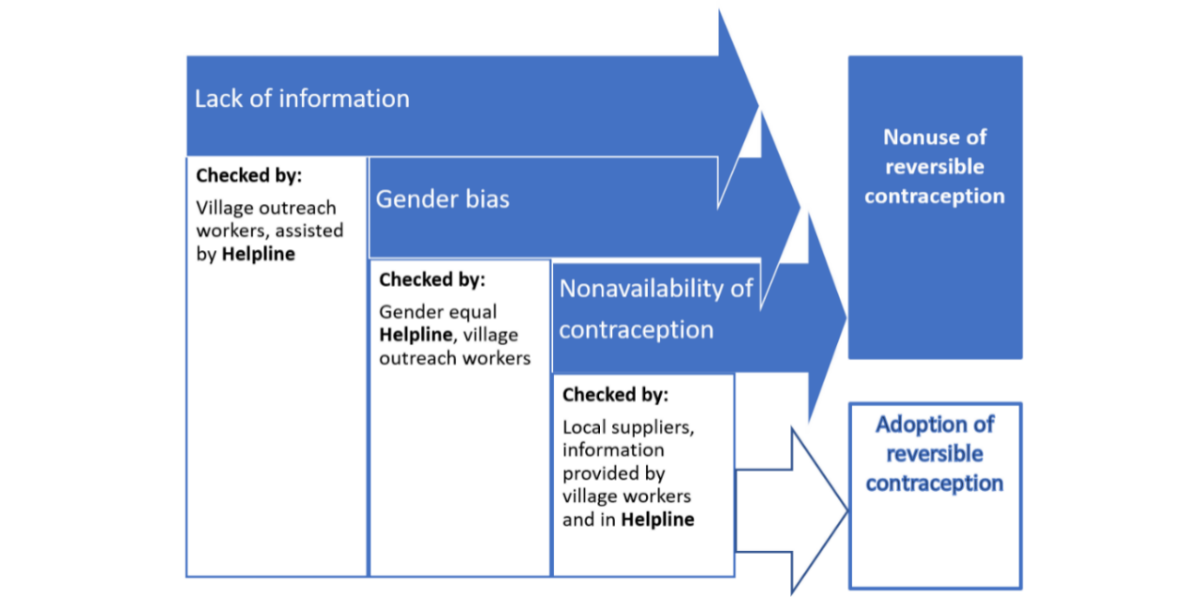
The role of the helpline in the intervention’s theory of change (hindrances in dark arrows and intervention activities in white boxes).

The intervention was based on the theory of change in a situation, when a woman or a couple was motivated to delay or avoid pregnancy, as expressed in Figure 1. On the basis of desk research and grassroots activities in another area, the project identified the following three main hindrances to the adoption of reversible contraceptive methods in a situation when contraception was in demand for a woman or couple: lack of information, gender bias, and unavailability or inaccessibility of contraceptives. Lack of information was to be addressed by providing a mobile helpline and marketing its services, gender bias was to be addressed by providing the helpline, which was an easily accessible source of support and information for the young and the women irrespective of elders’ and men’s acceptance, and finally, the supply of contraception issue was addressed in the mobile helpline by providing information on the local points of access to condoms, pills, and coils. However, the mobile helpline had to be supported by village workers and could not be implemented without initial personal contact with locals.

### The Control Area

The control site was a PHC area in Nashik, an adjacent district. This area was serviced by similar governmental maternal and health care services, including family planning services. For several years, there was a mother and child health program being implemented by an NGO, whose local village workers supported governmental services. Thus, both areas were served by governmental public health, assisted by NGO-based services that involved personal contact with the local people. In the control area, there was no mobile helpline comparable with the one in the intervention area.

### Study Design

The study was a quasi-experimental study, a controlled before-and-after study as described by Reeves et al [23]. The study sample was derived from clusters, one of which was the intervention site, and the other was control site.

#### Before Study

**Figure 2:**
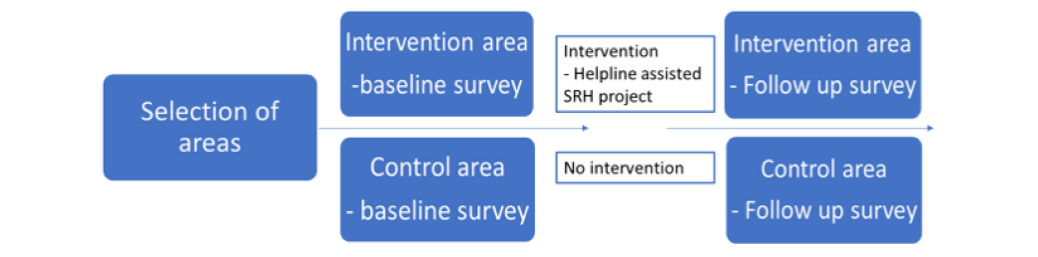
Study design. SRH: sexual and reproductive health.

An interview-based baseline survey was carried out from March to April 2015 in both the study areas to gather data on knowledge of, and practices related to, reversible contraceptive methods, needs related to SRH, and willingness to use mHealth services. The study design is shown in Figure 2.

#### After Study

To assess the outcome of the mobile helpline on SRH, a follow-up survey was carried out from November to December 2016. The follow-up survey was conducted in both areas about 18 months after the introduction of mHealth-assisted SRH services in the intervention area. The influence of external activities and information, such as government services and media programs toward family planning, was hypothesized to be similar in both areas. An NGO working with issues related to SRH was present in both areas but a mobile helpline was promoted only in the intervention area. Wider socioeconomic confounders were controlled using the control area. Hence, any differences in SRH observed between the areas after the intervention were analyzed as related to the potential outcome of the helpline intervention.

#### Study Sampling and Recruitment

The target sample size was 100 married men and 100 married women from each area. The intervention survey was intended to represent the area or cluster level and not the individual level, meaning that the study participants in the two survey rounds were not necessarily the same persons.

The sampling was carried out in two stages. A total of 10 villages (about 50% from each area) were randomly selected from each selected PHC using systematic sampling with a random start. A list of villages with a total population size of at least 500, which were arranged in ascending population sizes, served as the sampling frame from which sample selection was made. From each sampled village, 20 households were selected by a systematic sampling process using the left-hand rule, and 1 study subject was selected per household. If a household had more than 1 eligible subject, then one of them was chosen randomly. Trained research investigators were responsible for recruiting the study subjects. The inclusion criteria for selection of a subject were: age between 15 and 35 years, married, and lived permanently in the settlement. If the respondent was a visitor (eg, a daughter come down for delivery to her natal home or a visiting guest) then she was excluded from the study and the interview was terminated.

#### Measurements and Outcomes

This study used an interview-based data collection method, a structured questionnaire which suits low-literacy environments. The primary measurements were knowledge and practice of family planning, current use of contraception, intent for further use, and any changes in these after the intervention. The questionnaire included background socioeconomic and demographic information, questions on mobile phone ownership and use, union status and hygiene practices; knowledge, attitude, and practice of family planning; children and decision making, current use of contraception, intent for further use, and a contraceptive tracking sheet showing dynamics of contraceptive use of the study subjects. The data collection tool was first developed in English and then translated into the local language Marathi. A 3-day intensive training program was organized for 14 interviewers. A team of interviewers always included both a man and a woman. The interviews were conducted in privacy in the local (Marathi) language.

The main issue examined was whether a mobile helpline would improve people’s awareness of reversible contraceptive methods, and as a result of this would the use of such methods increase? The awareness of reversible contraception was operationalized by the question: *When you think of a family planning method, which is the first method that comes to your mind?*

The proportion of responses, other than female sterilization, was taken as a measure of awareness of reversible methods among the respondents. The respondents were also asked to give a list of all other contraceptive methods that they knew without being prompted. To examine the contraceptive prevalence and use of reversible methods, the respondent was first asked if she or he was currently doing anything to prevent or postpone her, or his wife’s, next pregnancy and then what they were currently doing to prevent it. The responses were coded as reversible methods (condom, oral contraceptive pills, IUCDs, and injectable contraceptives), permanent methods (female and male sterilization), and other methods (eg, withdrawal, abstinence, and herbs).

### Data Analysis

The intervention’s potential influence on the use of contraception based on the level of knowledge on contraceptive methods and SRH, access to contraception, and acceptability of mHealth support for SRH, were analyzed. The effect of intervention was estimated using logistic regression, adjusted for gender, by calculating robust standard errors to take into account clustering of individuals by the area (intervention or control). In each regression model, the effect of intervention was estimated by including a term for interaction between the intervention area and the period before and after the intervention. The exponent of the regression coefficient of the interaction term corresponding to the period after the intervention, along with the 95% CI, is reported here. The odds ratio for the control village multiplied by this exponent gives the odds ratio for the intervention village. The difference between the proportions after and before for each area is also reported. All analyses were performed with the statistical environment R (a free software environment supported by the R Foundation for Statistical Computing) [24].

### Ethical Considerations

The study was conducted in accordance with the Declaration of Helsinki of 1975, as revised in 2000, as well as with all applicable legal regulations governing data collection in India. The data did not contain any biological specimens but instead consisted of data on individual experiences, practices, attitudes, and knowledge, in addition to personal socioeconomic and demographic information. Informed consent was obtained in writing from every study subject after the nature and possible consequences of the study were explained. Participation was voluntary. All respondents were married. In the event of a respondent being below 18 years of age, consent was taken from a parent or spouse to include the underaged respondent in the study, with complete details of the study given to them, in addition to reading out the verbal consent form to the respondent.

The study was implemented in relation to the activities of two local NGOs [25] that gave their approval for the data collection. The study was approved by the local government authorities. As the study was carried out by independent scholars, the option of an institutional review board acceptance by a university was not available. Thus, an *ad hoc* ethical committee, consisting of three Indian members with expertise in social work and law and a social and health scientist, was formed with the help of the two NGOs to review the ethics of the study protocol and data collection plan. The ethical committee consented to the data collection and signed a statement of their approval.

## Results

### Descriptive Statistics

Table 1 presents background information of the surveys and respondents.

**Table 1:** Descriptive characteristics (source: baseline and follow-up surveys in two public health center areas in Maharashtra).

Characteristics			Intervention area	Control area
**Sample size, n**				
	**Baseline survey**			
		Women	103	100
		Men	102	100
		Total	205	200
	**Follow-up survey**			
		Women	90	101
		Men	88	101
		Total	178	202
**Respondent age (years), median (range), baseline survey**				
	Women		26 (24-30)	26 (22-30)
	Men		29 (26-34)	29 (26-33)
**Respondent age (years), median (range), follow-up survey**				
	Women		27 (25-31)	27 (23-31)
	Men		30 (27-35)	30 (27-34)
**Age at marriage (years), median (range), baseline survey**				
	Women		19 (13-28)	18 (14-30)
	Men		22 (17-30)	21 (16-29)
**Age at marriage (years), median (range), follow-up survey**				
	Women		19 (15-28)	18 (12-28)
	Men		23 (17-30)	22 (18-29)
**Literacy^a^ rate, n (%)**				
	Women^b^		87 (84.4)	76 (74.5)
	Men^b^		86 (84.3)	90 (90.0)
**Disadvantaged group, n (%)^b^**				
	Scheduled tribes and scheduled castes		132 (64.3)	150 (75.4)
	Other backward classes		45 (22.0)	34 (17.1)
**Access to mobile phone in household, n (%)^c^**				
	Women		87 (84.5)	93 (93.0)
	Men^c^		81 (79.4)	86 (86.0)

^a^Able to both read and write, according to own statement.

^b^Information collected in baseline survey only.

^c^Having at least one mobile phone in household.

A total of 405 married men and women aged 15 to 35 years participated in the baseline survey before the intervention, of which 200 were from the control area. Gender-wise distributions are presented in Table 1. In the postintervention survey, the total number of participants was 380, of which 202 were from the control area. 87.3% (152/179) and 50.0% (101/202) of the participants were in both the surveys of intervention and control areas, respectively.

The median age of men and women was 29 and 26 years, respectively, in both areas before the intervention. It was 30 and 27 years, respectively, during the follow-up survey (detailed descriptive tables are available in [26]). The quartile age ranges for men and women in the baseline survey were 26 to 34 and 22 to 30, respectively. Median age at marriage for women was 18 years. Most men (84.4%; 87/103) and women (84.3%, 86/102) in the intervention area could read and write, whereas slightly more men (90.0%; 90/100) and somewhat less women (74.5%, 76/102) could do so in the control area. Literacy was recorded as reported by the study subjects; it was recorded separately for reading and writing, and it did not differ significantly for men and women.

In both areas, the caste composition was characterized by a high proportion of socially disadvantaged populations. Both areas have atypically high proportions of tribal populations, and in the Thane district, the intervention area, the proportion is even higher than in Nashik. Scheduled caste (formerly called the untouchables) and scheduled tribe populations are the most disadvantaged social categories in India and their combined proportion was 64.3% (132/205) in the intervention area, and 75.4% (150/199) in the control area. In both areas, the main occupation was farming and unskilled labor. A majority of the respondents in both areas reported having access to a mobile phone, with only 21.6% (21/102) of men and 15.5% (16/103) of women reporting not having a mobile phone in the household in the intervention area, and even less in the control area, with 14.0% (14/100) of men and 7.0% (7/100) of women reporting similarly. The difference between the proportion of men (*P*=.30) and women (*P*=.10) having access to mobile phones in the two study areas was not significant. The fact that women appear to have slightly more access to mobile phones than men seems somewhat surprising.

The summary statistics of the survey responses (n [%] for men, women, and all) are provided in Table 2. Both areas manifested a considerable increase in the general awareness of contraception: in the follow-up survey, 75.8% (135/178) of respondents in the intervention area and 81.6% (160/196) in the control area were aware of means to avoid pregnancy. The proportion of those who had heard of a contraceptive method from an NGO rose in the intervention area from 5.0% (6/121) to 33.5% (59/176), whereas in the control area, it was 1.7% (2/118) before and 1.2% (2/172) after. The proportion of respondents using reversible contraception rose in both areas.

**Table 2:** Knowledge on contraceptive methods and sexual and reproductive health, access to contraception, and acceptability of mobile health support for sexual and reproductive health by study areas before and after the intervention (source: baseline and follow-up surveys, two primary health care areas in Maharashtra).

Outcome		Intervention area, n (%)		Control area, n (%)	
		Before	After	Before	After
**Has heard of any contraception method**					
	Men	49 (48.0)	76 (86.4)	46 (46.0)	85 (84.2)
	Women	72 (69.9)	59 (65.6)	72 (72.0)	75 (78.9)
	All	121 (59.0)	135 (75.8)	118 (59.0)	160 (81.6)
**Has heard of contraception method from NGO^a^**					
	Men	3 (6.1)	16 (18.6)	2 (4.8)	2 (2.2)
	Women	3 (4.2)	43 (47.8)	0 (0.0)	0 (0.0)
	All	6 (5.0)	59 (33.5)	2 (1.7)	2 (1.2)
**Reversible method first mentioned^b^**					
	Men	28 (59.6)	5 (6.8)	24 (52.2)	38 (46.3)
	Women	22 (32.4)	14 (21.9)	43 (67.2)	36 (48.0)
	All	49 (42.6)	19 (13.8)	67 (60.9)	74 (47.1)
**Uses contraception now**					
	Men	30 (29.4)	54 (61.4)	54 (54.0)	25 (24.8)
	Women	59 (57.3)	37 (41.1)	69 (69.0)	55 (57.9)
	All	89 (43.4)	91 (51.1)	123 (61.5)	80 (40.8)
**Using reversible method^c^**					
	Men	8 (26.7)	22 (40.7)	15 (27.8)	14 (56.0)
	Women	14 (23.7)	17 (45.9)	26 (37.7)	14 (25.5)
	All	22 (24.7)	39 (42.9)	41 (33.3)	28 (35.0)
**Willing to call sexual health helpline^d^**					
	Men	44 (56.4)	88 (100.0)	84 (95.5)	88 (91.7)
	Women	100 (100.0)	86 (95.6)	75 (81.2)	88 (94.7)
	All	144 (80.9)	174 (97.8)	159 (88.3)	176 (93.1)

^a^NGO: nongovernmental organization.

^b^First contraceptive method that comes to mind is a reversible method.

^c^Using some method other than sterilization, that is, a reversible method.

^d^Willing to call a male or female health worker to anonymously ask about sexual problems.

### The Intervention and Contraception

Table 3 shows the changes in the outcomes (proportions) in the intervention and control areas, and the exponent of the regression coefficient corresponding to the interaction of intervention area and the period after intervention, adjusted for gender. In terms of practice, the change in the direction toward reversible methods is evident in the intervention area. Both the current use of contraception (8% increase in the intervention area vs 21% decrease in the control area) and the use of reversible contraception (18% increase in the intervention area vs 2% increase in the control area) have increased in the intervention area compared with the control area.

The general level of awareness of reversible contraception, shown by the first contraceptive method that came to respondents’ mind being a reversible method, neither improved in the intervention area nor in the control area. On the contrary, fewer respondents in both areas mentioned a reversible method as the first method that came to their mind (decrease of 29% in the intervention area vs decrease of 14% in the control area). This means that, in the follow-up survey in both areas, it had become more common to mention female sterilization as the first method that came to mind. In the intervention area, the acceptability of contacting a helpline for SRH needs rose by 17% compared with the control area’s increase of 5%.

**Table 3:** Odds ratios (95% CIs) for interaction between intervention area and period after intervention and mean change for intervention versus control area on knowledge and practice of sexual and reproductive health (source: baseline and follow-up surveys, two primary health center areas in Maharashtra).

Outcome	Odds ratio (95% CI)	Change (after and before), intervention versus control area
Has heard of any contraception method	0.85 (0.844-0.855)	20 versus 23
Has heard of contraception method from NGO^a^	14.203 (13.265-15.208)	29 versus −1
Reversible method first mentioned^b^	0.363 (0.36-0.366)	−29 versus −14
Uses contraception now	3.207 (3.037-3.388)	8 versus −21
Using reversible method^c^	2.053 (1.856-2.271)	18 versus 2
Willing to call sexual health helpline^d^	6.038 (4.7-7.759)	17 versus 5

^a^NGO: nongovernmental organization.

^b^The first contraceptive method that comes to mind is a reversible method.

^c^Using method of contraception other than sterilization or traditional method, that is, a reversible method.

^d^Willing to call a male or female health worker to ask anonymously about sexual problems.

In the intervention area, 274 individuals aged between 15 and 52 years made a total of 964 calls to the mobile helpline on issues pertaining to SRH. This meant that repeat calls averaged around 3.5 calls per client. The typical questions dealt with were as follows:

- Information about family planning and contraceptive methods (their contraindications and side effects if any) and access to services (where, how far, and what cost): 31.0% (85/274) of calls.
- Sexual health issues such as itching in genitals, concerns regarding masturbation, wet dreams, condoms access, and use: 43.0% (118/274) of calls.
- Issues pertaining to maternal and child health (vaccination schedules, nutrition-related questions, and other related matters): 13.1% (36/274) of calls.
- Menstrual health and hygiene: 13.1% (36/274) of calls.

Questions on masturbation, condoms, and symptoms in genitals were mainly from men, and questions on menstrual hygiene were from adolescent girls. Questions on family planning and other contraceptive methods, other than condoms, came mainly from married women.

## Discussion

### Principal Findings

Both the current use of contraception and the use of reversible contraception have increased in the intervention area compared with the control area. Contraceptive knowledge increased in both areas, whereas fewer people first mentioned a reversible method in the follow-up survey than in the baseline survey, in both areas. Thus, the study suggests that the mobile helpline has had a bearing on the practice of contraception, whereas there is less evidence of effect on knowledge.

This apparent lack of improvement in awareness of reversible contraceptives might relate partly to the measurement used for the awareness: whether the first method that came to a respondent’s mind was a reversible method. In India, the universal connotation of family planning is female sterilization, a permanent method, which might turn out to be slow to change. Other types of questions to measure awareness of reversible methods might have painted a different picture. Moreover, this growing awareness of female sterilization compared with reversible methods in the follow-up survey might reflect governmental health service campaigns for female sterilization which the health workers were discussing in the communities in the months just before the follow-up survey, leading to an influence on the answers in the survey.

The study found that contraceptive use (both reversible methods and sterilization combined) at the time of the survey increased in the intervention site whereas it fell in the control site. This decrease found in the control area is rather unusual and needs further examination. In addition, the latest National Family Health Survey [21] showed a slight decline in the prevalence of family planning use among currently married women in Maharashtra. However, the decline witnessed in this study in the control area is more pronounced. It is unlikely that demand for family planning would have so strongly reduced within 18 months. It is possible that a temporary structural or administrative condition in the control area, such as reduced supply of contraceptives or absence of health care service personnel, could have caused a decline in contraceptive prevalence. However, this decrease in general contraceptive use (in practice, of female sterilization) in the control area does not explain away the increase in reversible contraception in the intervention area. Reversible contraception remained about as popular in the control area at both points of measurement, whereas in the intervention area the popularity of reversible contraception increased considerably. The adoption of female sterilization and reversible contraception are governed partly by different dynamics, with sterilization being a terminal method while reversible methods are mainly used for spacing births. This means that their developments are not necessarily interrelated. Consequently, the main result of the analysis, that the mobile SRH helpline is associated with increased use of reversible contraceptives, would not have to be compromised despite the decline in general contraceptive prevalence in the control area.

In both the intervention and in the control areas, NGOs working with issues related to SRH were present along with the standard local governmental services. The two NGOs provide basic information and services related to maternal and reproductive health to the relatively underprivileged populations of the two areas. The main difference between the two areas was the presence and promotion of a mobile helpline on SRH only existing in the intervention area. The results show that the intervention building upon a mobile helpline on SRH was associated with the increased use of reversible contraception compared with the control area. Although awareness of family planning generally strengthened, the awareness of reversible methods did not increase in the intervention area more than in the control area. This would imply that the governmental services and information channels have increased local people’s awareness on female sterilization in both areas.

A review of studies dealing with the impact of interventions that aim at influencing knowledge, attitudes, beliefs, and discussions regarding family planning and in increasing contraceptive use found that impacts are often a result of programs that have considered the importance of varied approaches to reaching women and couples [27]. The intervention examined here manifested this approach by including face-to-face activities, an anonymous helpline, and support to contraceptive availability in local settings.

The helpline received acceptance in the intervention area so that a 17–percentage point rise in the acceptability of such service provided by an NGO in case of SRH problems was evident. Villagers and helpline customers had expressed their appreciation of the fact that they could call whenever it was a convenient time and place for them. Most rural families in the project area, as in much of India, share a phone so that it stays at home and many family members can use it [20]. The need to consider mHealth interventions from a relational perspective, not only as the choice of an individual, becomes essential here [28]. Thus, a mobile helpline that can be confidentially approached at a time most convenient to the client is essential for a successful mHealth service. Clients particularly preferred a voice-based personal service, as they felt insecure about the terminology on issues of SRH. Many could not write or read fluently in the local language, Marathi. The experiences from this mHealth intervention study point to the need for program developers and designers to explore contextual and implementation factors seriously [6].

A considerable proportion of the questions raised in the mobile helpline touched on sexuality and issues other than contraception. There is clearly a great need in the area to provide more general SRH services and information than only family planning services to the local population, as well as to men and adolescents. A comprehensive SRH approach works better than a narrow family planning approach in winning local people’s confidence, which is essential for sustainable results.

It was evident from the intervention that among rural, socioeconomically underprivileged populations, women and men have an unmet need for reversible family planning methods [29]. Although the governmental programs have started to pay more attention to contraceptive choices instead of sole reliance on female sterilization, there is still a long way to go. This intervention study shows that services that integrate mHealth in a context-sensitive way to face-to-face health care services can provide better results in rural India and assumedly also in other contexts in less developed societies.

### Strengths and Limitations

This study was a rare attempt to examine the outcome of an mHealth intervention by making use of a control area. However, the conclusions would have been stronger if the samples would have been larger and the time frame would have been longer. The outcome was assessed after only half a year after the end of the intervention, which makes it difficult to say much about the perseverance of the changes. Changes in perceptions take a longer time to emerge in a measurable form.

The apparent inconsistency in the intervention area findings, in that knowledge of reversible methods seems to have fallen but the use of reversible contraception nevertheless increased substantially, can point to the problems in the instrumentalization of knowledge on reversible methods. Relying on the first thing that comes to mind question may have rendered the study vulnerable to a government campaign on female sterilization. If another measure of knowledge on reversible contraception were used this apparent inconsistency might have disappeared. The study design was neither a true panel design (interviewing the same respondent both in the baseline and the follow-up surveys) nor purely a successive cross-sectional design. Of all the respondents in the follow-up survey in the intervention area, 85% had also been interviewed in the baseline, whereas this proportion was 50% in the control area, meaning that a larger proportion of respondents in the intervention than control area had been interviewed earlier. This unplanned asymmetry was an outcome of some unforeseen practical imperatives in the field study. However, this fact has had only a minor influence on the age range of the respondents that appears nearly similar in the follow-up survey in the two areas. We do not see any other logic behind how this partial asymmetry in study design would have significantly influenced the results of the logistic regression analysis. The intervention implemented in the study was a community-level intervention and not an individual-level intervention, as the entire villages were targeted by the intervention. This is the main reason why it does not make a difference if the baseline subjects differ from the postintervention subjects. We are interested in population-level changes, and these can be studied by having different subjects pre and postintervention.

## Abbreviations

IUCD: intrauterine contraceptive device
mHealth: mobile health
NGO: nongovernmental organization
PHC: primary health center
SRH: sexual and reproductive health

## References

[1] Shet A, Arumugam K, Rodrigues R, Rajagopalan N, Shubha K, Raj T, D'souza G, de Costa A (2010). Designing a mobile phone-based intervention to promote adherence to antiretroviral therapy in South India. AIDS Behav.

[2] (2011). World Health Organization.

[3] Mascarenhas A (2014). The Indian Express.

[4] Ilozumba O, Dieleman M, Kraamwinkel N, van Belle S, Chaudoury M, Broerse JE (2018). 'I am not telling. The mobile is telling': factors influencing the outcomes of a community health worker mhealth intervention in India. PLoS One.

[5] Reynolds NR, Satyanarayana V, Duggal M, Varghese M, Liberti L, Singh P, Ranganathan M, Jeon S, Chandra PS (2016). MAHILA: a protocol for evaluating a nurse-delivered mhealth intervention for women with HIV and psychosocial risk factors in India. BMC Health Serv Res.

[6] Lerma K, Reyes G, Tiwari S, Tewari A, Hastings C, Blumenthal PD (2018). Acceptability of a text message-based fertility awareness application for family planning in Lucknow, India. Int J Gynaecol Obstet.

[7] Majumdar A, Kar SS, Kumar G, Palanivel C, Misra P (2015). mHealth in the prevention and control of non-communicable diseases in India: current possibilities and the way forward. J Clin Diagn Res.

[8] Smith C, Gold J, Ngo TD, Sumpter C, Free C (2015). Mobile phone-based interventions for improving contraception use. Cochrane Database Syst Rev.

[9] National Family Health Survey.

[10] (2013). National Health Mission.

[11] de Oliveira IT, Dias JG, Padmadas SS (2014). Dominance of sterilization and alternative choices of contraception in India: an appraisal of the socioeconomic impact. PLoS One.

[12] Raj A, Ghule M, Ritter J, Battala M, Gajanan V, Nair S, Dasgupta A, Silverman JG, Balaiah D, Saggurti N (2016). Cluster randomized controlled trial evaluation of a gender equity and family planning intervention for married men and couples in rural India. PLoS One.

[13] Char A, Saavala M, Kulmala T (2011). Assessing young unmarried men's access to reproductive health information and services in rural India. BMC Public Health.

[14] Char A, Saavala M, Kulmala T (2010). Influence of mothers-in-law on young couples' family planning decisions in rural India. Reprod Health Matters.

[15] Rimal RN, Sripad P, Speizer IS, Calhoun LM (2015). Interpersonal communication as an agent of normative influence: a mixed method study among the urban poor in India. Reprod Health.

[16] Diamond-Smith N, Campbell M, Madan S (2012). Misinformation and fear of side-effects of family planning. Cult Health Sex.

[17] Samal J, Dehury RK (2015). Family planning practices, programmes and policies in India including implants and injectables with a special focus on Jharkhand, India: a brief review. J Clin Diagn Res.

[18] Calhoun LM, Speizer IS, Rimal R, Sripad P, Chatterjee N, Achyut P, Nanda P (2013). Provider imposed restrictions to clients' access to family planning in urban Uttar Pradesh, India: a mixed methods study. BMC Health Serv Res.

[19] (2018). Telecom Regulatory Authority of India, Government of India.

[20] Tenhunen S (2018). A Village Goes Mobile: Telephony, Mediation, and Social Change in Rural India.

[21] National Family Health Survey.

[22] Data for All.

[23] Reeves BC, Wells GA, Waddington H (2017). Quasi-experimental study designs series-paper 5: a checklist for classifying studies evaluating the effects on health interventions-a taxonomy without labels. J Clin Epidemiol.

[24] R project.

[25] U-Respect Foundation.

[26] (2018). Akra-Numero.

[27] Mwaikambo L, Speizer IS, Schurmann A, Morgan G, Fikree F (2011). What works in family planning interventions: a systematic review. Stud Fam Plann.

[28] Nahar P, Kannuri N, Mikkilineni S, Murthy G, Phillimore P (2017). mHealth and the management of chronic conditions in rural areas: a note of caution from southern India. Anthropol Med.

[29] Tapare VS, Parande MA, Borle PS (2017). Unmet need for contraception among married women of reproductive age in rural Maharashtra. Int J Community Med Public Health.

